# The impact of a livelihood program on depressive symptoms among people living with HIV in Cambodia

**DOI:** 10.3402/gha.v9.31999

**Published:** 2016-08-09

**Authors:** Mayumi Shimizu, Siyan Yi, Sovannary Tuot, Samedy Suong, Samrithea Sron, Akira Shibanuma, Masamine Jimba

**Affiliations:** 1 Department of Community and Global Health, Graduate School of Medicine, The University of Tokyo, Tokyo, Japan; 2 KHANA Center for Population Health Research, Phnom Penh, Cambodia; 3 KHANA Social Enterprise, Phnom Penh, Cambodia

**Keywords:** people living with HIV, livelihood program, depressive symptoms, quasi-experimental nonequivalent comparison group study, Cambodia

## Abstract

**Background:**

Psychological and social problems are major concerns in this era of successful antiretroviral therapy. Although livelihood programs have been implemented extensively to improve the daily living conditions of people living with HIV in Cambodia, no studies have yet investigated the impacts of these programs on the mental health of this vulnerable population. Therefore, we examined the impact of a livelihood program on depressive symptoms and associated factors among people living with HIV in Cambodia.

**Design:**

A quasi-experimental, nonequivalent comparison group study was conducted in six provinces of Cambodia in 2014. Data were collected from an intervention group comprising 357 people living with HIV who had participated in the livelihood program and a comparison group comprising 328 people living with HIV who had not participated in this program. Multiple logistic regression analysis was carried out to examine the association between livelihood-program participation and depressive symptoms as measured by the depressive symptoms subscale of the 25-item Cambodian version of the Hopkins Symptom Checklist. A propensity score matching was used to examine the effect of the livelihood program on depressive symptoms while controlling for selection bias.

**Results:**

Overall, 56.0% and 62.7% of the participants in the intervention and comparison groups, respectively, met the Hopkins Symptom Checklist threshold for depressive symptoms. The multiple logistic regression analysis showed that the participants in the intervention group had significantly lower odds of having depressive symptoms (adjusted odds ratio 0.68, 95% confidence interval 0.52–0.88). The analysis from propensity score matching indicated that the livelihood program helped mitigate depressive symptoms among the participants in the intervention group (T=−1.99).

**Conclusions:**

The livelihood program appeared to help mitigate the burden of depressive symptoms among people living with HIV in Cambodia. Thus, this program should be scaled up and modified to better improve participants’ mental health.

## Introduction

Although much progress has been made in HIV prevention and care, psychological symptoms and social problems remain a major concern for people living with HIV on antiretroviral therapy (ART) (1, 2). Depression is reportedly the most prevalent mental health problem among people living with HIV (3); indeed, they are two to four times more likely to be diagnosed with depression than populations without HIV (4, 5). Moreover, depression is associated with poor adherence to ART (6, 7), more rapid progression of HIV (8, 9), and a lower quality of life (10, 11).

There is a vicious cycle between HIV infection and poverty, wherein HIV-affected households are more likely to develop economic problems, which in turn worsen individuals’ symptoms and so on (12). Poverty is a multidimensional construct, of which a particularly important dimension is food insecurity, a condition that has been linked with poorer adherence to ART and HIV treatment outcomes (13, 14) and increased mortality among people living with HIV (15, 16). Using only medical approaches is insufficient for supporting people living with HIV who are dealing with these multifaceted problems (12). Therefore, several countries have implemented livelihood programs for people living with HIV to improve their economic conditions and food security (17, 18). A randomized controlled trial in Kenya has noted that livelihood programs improve HIV clinical outcomes and reduce food insecurity among people living with HIV (18, 19). Livelihood programs largely comprise income-generating activities such as small grants, village savings and loan associations, crop production or animal husbandry, provision of agriculture materials, training in agricultural skills and techniques, and development of small businesses (17, 20, 21).

Mental health is becoming an increasingly important issue in Cambodia (22). A Cambodian mental health survey revealed high rates of a number of serious mental health problems throughout the country, such as child abuse, suicidal ideation, and alcohol problems (23). Furthermore, among the general population, the prevalence of depressive symptoms and anxiety was estimated to be 16.7% and 27.4%, respectively (23). However, as is the case in many low- and middle-income countries (24, 25), mental health remains a low priority for the Cambodian government – specifically, it has less than 1.0% of the total health budget (23). Cambodia lacks any independent psychiatric institutions and, more shockingly, possesses only 14 psychiatric beds for the entire population (22). Depressive symptoms have been assessed among various Cambodian populations, including the general population (26, 27), refugees, immigrants (28, 29), and landmine survivors (30); however, little is known about depressive symptoms among people living with HIV in Cambodia (31).

Cambodia has faced a serious HIV epidemic since the first case was reported in 1991 (32). However, due to the efforts of HIV prevention programs, care, and treatment, the estimated HIV prevalence among adults aged 15–49 years declined from its peak of 1.7% in 1998 to 0.7% in 2014 (32, 33). In more concrete terms, with the success of the prevention of HIV infection and the marked improvement of ART coverage, the estimated number of people living with HIV, among adults aged 15 years and over, decreased from its peak of 102,440 in 2000 to 72,159 in 2014 (32). The estimated number of AIDS deaths declined from its peak of 6,657 in 2004 to 2,321 in 2014 (32). Additionally, the estimated number of new HIV infections fell from its peak of 20,978 in 1995 to 1,003 in 2014 (32, 33).

Despite these improvements, a diagnosis of HIV continues to have a considerable negative socioeconomic impact on people living with HIV and their families. HIV-affected households have a lower income per capita and are 1.7 times as likely to live below the poverty line as non-HIV-affected households (34). Additionally, HIV-affected households in Cambodia are more likely to experience food insecurity, with over half receiving food support compared to only 4.0% of non-HIV-affected households (34). To improve the living conditions of people living with HIV, a livelihood program was implemented in Cambodia in 2010. However, no studies have yet assessed the psychological impacts of this livelihood program on program participants. Therefore, the aim of this study was to examine the impact of this livelihood program on depressive symptoms and associated factors among people living with HIV in Cambodia.

## Methods

### Study design

This quasi-experimental, non-equivalent comparison group study was conducted in Cambodia in collaboration with a large national non-governmental organization (NGO), KHANA, as part of a larger evaluation study of the ongoing KHANA livelihood program.

### KHANA livelihood program

KHANA has provided integrated HIV prevention, care, and support services at the community level since 1996. In 2014, along with several community-based NGOs as implementing partners, KHANA covered 17,610 people living with HIV in 22 provinces in Cambodia (35). Since KHANA created its livelihood program in 2010, the program has expanded into 13 provinces (as of 2014). The objectives of this livelihood program are as follows: 1) to mitigate the vulnerability and address the current socioeconomic needs of households infected and affected by HIV; 2) to empower households with the necessary skills to produce sufficient nutritional foods; and 3) to enhance households’ capacity to generate regular incomes (36).

KHANA's livelihood program offers support to people living with and affected by HIV through a variety of activities, including encouraging the formation of village savings and loan associations and providing skills training, small cash grants, and ongoing technical support. Village savings and loan associations are groups of 10–30 members of a community who pool their funds to offer loans to their members for a variety of reasons, including emergencies, basic household needs, purchasing food, school fees for children, travel costs to ART sites, and capital for small businesses (37). Each association member generally contributes between 0.75 and 1.25 USD per month and is allowed to borrow up to three times their share, although they must repay what they are lent inclusive of interest within 3 months (37). The skills training covered the following topics: agricultural techniques including vegetable or rice farming, animal husbandry, and fish farming; resource management (water, land, livestock, and human); and financial and business management (38). The small cash grants provide recipients with up to 120 USD as startup capital for income-generating activities (39).

### Data collection

Data were collected in six provinces: Battambang, Kampong Cham, Kampong Speu, Pursat, Siem Reap, and Takeo from August to September 2014. We used a two-stage sampling method to select the study sample. First, we selected the provinces that would serve as study sites based on the length of their implementation of the livelihood program and its accessibility. We then recruited the sample using KHANA's database of people living with HIV in these six provinces: specifically, 981 people living with HIV were identified as potential intervention group members and 5,549 as potential comparison group members. Second, we selected health centers, which served as the sampling unit, because people living with HIV were listed according to the health center at which they were treated in the database. Health centers were categorized according to the presence of the KHANA livelihood program, which resulted in two lists of health centers: those with the livelihood program (i.e. the intervention group) and those without (i.e. the comparison group). Health centers with more than 10 people living with HIV registered were selected from each list for the intervention and comparison groups to obtain the required sample size in each province. KHANA's collaborating local NGOs contacted and recruited people living with HIV for both groups.

**Fig. 1:**
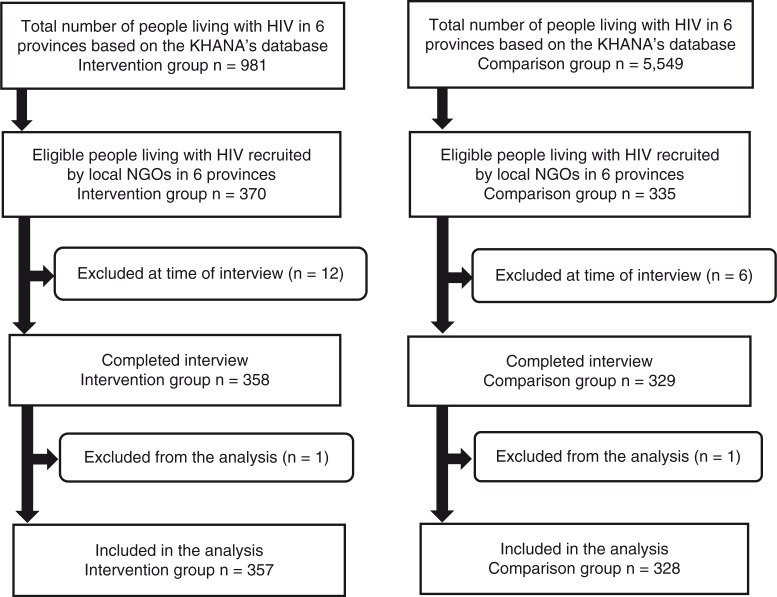
Study participant flowchart.

To be included in the study, a person had to 1) be HIV seropositive and have been living with HIV for more than 1 year since diagnosis; 2) be aged 18 years or older; 3) understand the Khmer language; 4) have been a resident in the study area for more than 1 year; 5) give written informed consent for voluntary participation; and 6) for the intervention group, have been enrolled in the livelihood program for at least 1 year. All the identified people living with HIV who met the inclusion criteria at the selected health centers were considered eligible for participation. Ultimately, data from 357 people living with HIV in the intervention group and 328 people living with HIV in the comparison group were used for analyses. All study participants were administered a structured questionnaire in Khmer through face-to-face interviews by trained interviewers in private settings (Fig. 1).

## Measures

### Depressive symptoms

Depressive symptoms were measured using the depressive symptoms subscale of the 25-item Cambodian version of the Hopkins Symptom Checklist (HSCL-25) (40). This subscale comprises 15 items. The HSCL-25 was developed as a screening tool for measuring symptoms of anxiety and depression (30, 40). It has been used in research on various populations worldwide, including patients in family practice and family planning services (41, 42), refugees and immigrants (28, 29), people living with HIV (43–47), and the Cambodian general population (26, 27). The Cambodian version of the HSCL-25 was developed as one of the Indochinese versions (40).

Each item is rated on a four-point Likert-type scale ranging from 1 (not at all) to 4 (extremely). The total score for depressive symptoms is calculated by averaging the item scores. Using the cutoff established among the Cambodian population (26), individuals with scores greater than 1.75 were considered symptomatic (40). The inter-rater reliability coefficients for the total, anxiety, and depressive symptoms subscales were higher than 0.98 (40). The depressive symptoms subscale also demonstrated good internal consistency (Cronbach's alpha=0.86) in the Cambodian mental health survey (23). In the present study, the Cronbach's alpha was 0.85.

### Food insecurity

Food insecurity was assessed by using the Household Food Insecurity Access Scale. This nine-item scale was developed as part of the US Agency for International Development's food and nutrition technical assistance project and has been validated in several countries, including Cambodia (48–50). The scale covers several domains relating to food insecurity, such as ‘anxiety and uncertainty about the household food supply’, ‘insufficient food quality’, and ‘insufficient food intake and its physical consequences’ (48). Each item has a binary (yes or no) and a frequency (rarely, sometimes, or often) response option concerning occurrence in the past 4 weeks (48). The frequency option is only available to participants who answer ‘yes’ to the binary option (48). Typically, participant households are classified into four levels of food insecurity: food secure and mildly, moderately, and severely food insecure (48). However, a dichotomous variable (food secure vs. food insecure) was used for the multiple logistic regression analysis (51). The Household Food Insecurity Access Scale has reported good internal consistency (Cronbach's alpha=0.91–0.97) in several studies on people living with HIV in African countries (51, 52). The Cronbach's alpha for this measure was 0.77 in the present study.

### Other covariates

Sociodemographic, HIV-related, and psychosocial variables were assessed as covariates. These included program participation status, age, gender, education, marital status, monthly household income per capita, debt, ART status, length of time since HIV seropositive diagnosis, receipt of food assistance, internalized HIV-related stigma, and self-esteem. We calculated monthly household income per capita by transforming the monthly household income from Cambodian riel into USD (with an exchange rate of 4,050 Cambodian riel=1 USD as of August 1, 2014) and then dividing by household size. The six-item Internalized AIDS-Related Stigma Scale was used to measure HIV-related internalized stigma (53). Each item is rated dichotomously (1=agree or 0=disagree), and scores are summed to form a total score ranging from 0 to 6. Higher total scores indicate greater internalized HIV-related stigma (53). A dichotomous variable was created for the multiple logistic regression analysis according to a median split (3): participants with scores of 0–2 were considered to have low levels of internalized HIV-related stigma, while those with scores of 3–6 were considered to have high levels (54). In this study, the Cronbach's alpha was 0.67.

Self-esteem was measured using the 10-item Rosenberg Self-Esteem Scale (55, 56). Each item on this scale is scored on a four-point Likert scale ranging from 4 (strongly agree) to 1 (strongly disagree). Negative items are reverse scored and range from 1 (strongly agree) to 4 (strongly disagree). Total scores are derived by summing all 10 items and range from 10–40, with higher scores indicating greater self-esteem. We categorized participants according to whether they were lower or higher than the mean score (28.7): participants with a score of less than 28.7 were categorized as having low self-esteem, and those with a score of 28.7 or more were considered to have high self-esteem. This was done to decrease the effects of outliers and facilitate explanation of the results in the multiple logistic regression analysis. In this study, the Cronbach's alpha was 0.57.

## Statistical analyses

Descriptive analyses were conducted for the sociodemographic and other characteristics of the study sample. Multiple logistic regression analysis was then carried out to examine the association between participation in the livelihood program and depressive symptoms. The abovementioned covariates were included in the analysis. These covariates were selected based on the findings of previous studies. The multiple logistic regression analysis was estimated using cluster-robust standard errors to control for biases caused by the differences between provinces.

To further examine the effect of the livelihood program on depressive symptoms, we also conducted a propensity score matching. Propensity scores were calculated using a logistic regression model adjusted for participants’ basic characteristics: namely, age, gender, education, marital status, monthly household income per capita, debt, ART status, length of time since HIV seropositive diagnosis, and receipt of food assistance. Kernel matching was used to match the participants of the livelihood program with non-participants of the program. Specifically, we also matched all of the participants of the livelihood program with the weighted average of non-participants using weights that were inversely proportional to the distance between the propensity scores of these two groups. The average treatment effect on the treated was estimated using the matched sample. To check the balance after matching, standardized percentage bias and Rubin's B and R were examined. All statistical tests were two-sided with a significance level of *p*<0.05 and were performed using STATA SE Version 12 for Windows.

## Ethics statement

The study protocol was approved by both the National Ethics Committee for Health Research at the Cambodian Ministry of Health and the Research Ethics Committee of the Graduate School of Medicine at the University of Tokyo in Japan. Written informed consent was obtained from all study participants before the interviews. Confidentiality was maintained by using non-identifying numbers throughout the study for all instruments and analyses.

## Results

### Participant characteristics

Among the 357 intervention group participants, 46.8% had participated in the livelihood program for between 1 and less than 2 years, and 53.2% had participated for between 2 and 4 years. The mean age of the intervention group was 44.5 years (standard deviation [SD]=7.9), while the mean age of the comparison group (*n*=328) was 43.4 years (SD=8.7). The proportion of women was significantly higher in the intervention group than in the comparison group (73.7% vs. 62.5%, *p=*0.002). In both groups, around 80% of the study participants had at least some formal education, and more than half of the participants were married or cohabiting with a partner. The median monthly household income per capita was 21.6 USD (interquartile range [IQR]=10.4, 37.0) for the intervention group, and 18.5 USD (IQR=7.9, 34.6) for the comparison group. Over 70% of the study participants in both groups reported having debt during the past 12 months. The participants in the intervention group were significantly less likely to have received food assistance compared to those in the comparison group (38.1% vs. 46.0%, *p=*0.035). In both groups, 98% of the participants were on ART at the time of the survey, and the median time since HIV seropositive diagnosis was 8 years (intervention group: IQR=6, 10; comparison group: IQR=5, 10). In both groups, around 60% of the participants reported high Internalized AIDS-Related Stigma Scale scores. The prevalence of food insecurity was 91.6% in the intervention group and 94.8% in the comparison group (Table 1).

**Table 1:** Background characteristics of study participants

Characteristic	Intervention group *n*=357 (%)	Comparison group *n=*328 (%)	Total *n*=685 (%)	*p*
Age (in years; range: 18–73)				
Mean (SD)	44.5 (7.9)	43.4 (8.7)	44.0 (8.3)	0.095
<44	177 (49.6)	166 (50.6)	343 (50.7)	0.820
≥44	180 (50.4)	146 (49.4)	342 (49.3)	
Gender				
Men	94 (26.3)	123 (37.5)	217 (31.7)	**0.002**
Women	263 (73.7)	205 (62.5)	468 (68.3)	
Formal education				
No	71 (19.9)	68 (20.7)	139 (20.3)	0.784
Yes	286 (80.1)	260 (79.3)	546 (79.7)	
Marital status				
Divorced/separated/widowed/single	154 (43.1)	123 (37.5)	277 (40.4)	0.133
Married/cohabitating	203 (56.9)	205 (62.5)	408 (59.6)	
Monthly household income per capita^a^ (in USD; range: 0–926)				
Median (IQR)	21.6 (10.4, 37.0)	18.5 (7.9, 34.6)	20.6 (9.9, 37.0)	0.056
<20.6	174 (48.7)	172 (52.6)	340 (50.6)	0.313
≥20.6	183 (51.3)	158 (47.4)	344 (49.4)	
Debt in the past 12 months				
No	91 (25.5)	92 (28.1)	183 (26.7)	0.450
Yes	266 (74.5)	236 (71.9)	502 (73.3)	
Food assistance				
No	221 (61.9)	177 (54.0)	398 (58.1)	**0.035**
Yes	136 (38.1)	151 (46.0)	287 (41.9)	
ART status				
Not receiving ART	8 (2.2)	7 (2.1)	15 (2.2)	0.924
Receiving ART	349 (97.8)	321 (97.9)	670 (97.8)	
Time since HIV seropositive diagnosis^a^ (in years; range: 1–22)				
Median (IQR)	8 (6, 10)	8 (5, 10)	8 (5, 10)	0.575
<8	152 (42.7)	151 (46.0)	303 (44.3)	0.380
≥8	204 (57.3)	177 (54.0)	381 (55.7)	
Internalized HIV-related stigma^b^ (range: 0–6)				
Median (IQR)	3 (1, 4)	3 (2, 4)	3 (2, 4)	0.056
Low (IA-RSS score 0–2)	147 (41.4)	128 (39.0)	275 (40.3)	0.526
High (IA-RSS score 3–6)	208 (58.6)	200 (61.0)	408 (59.7)	
Self-esteem score^c^ (range: 15–38)				
Mean (SD)	28.7 (3.3)	28.6 (3.5)	28.7 (3.4)	0.740
Low (RSES score<28.7)	162 (45.6)	159 (48.8)	321 (47.1)	0.412
High (RSES score≥28.7)	193 (54.4)	167 (51.2)	360 (52.9)	
Household food insecurity^b^				
Food secure	30 (8.4)	17 (5.2)	47 (6.9)	0.096
Food insecure	326 (91.6)	310 (94.8)	636 (93.1)	
Depressive symptoms (HSCL-25)^a^ (range: 1–3.73)				
Median (IQR)	1.87 (1.47, 2.27)	1.93 (1.60, 2.40)	1.93 (1.53, 2.33)	**0.046**
No (scores for depressive symptoms≤1.75)	157 (44.0)	122 (37.3)	279 (40.8)	0.076
Yes (scores for depressive symptoms>1.75)	200 (56.0)	205 (62.7)	405 (59.2)	
Length of program participation (in years; range: 1–4)				
Median (IQR)	2 (1, 2)			
1 to <2	167 (46.8)			
2 to <3	126 (35.3)			
≥3	64 (17.9)			
Participation in the program activities				
Village savings and loan association	347 (97.2)			
Skills training	176 (49.3)			
Small cash grant	117 (32.8)			

IQR, interquartile range; SD, standard deviation; ART, antiretroviral therapy; HSCL-25, 25-item Hopkins Symptom Checklist; IA-RSS, Internalized AIDS-Related Stigma Scale; RSES, Rosenberg Self-Esteem Scale. Bold denotes statistically significant *p*-value at 5% level.

a One participant did not respond to this item.

b Two participants did not respond to this item.

c Four participants did not respond to this item.

### Prevalence rates of depressive symptoms

The median scores of the HSCL-25 depressive symptoms subscale were 1.87 (IQR=1.47, 2.27) in the intervention group and 1.93 (IQR=1.60, 2.40) in the comparison group. The scores differed significantly between the groups (*p=*0.046). A majority of the study participants (56.0% of the intervention group and 62.7% of the comparison group) met the HSCL-25 threshold for depressive symptoms (score of greater than 1.75; Table 1).

### Factors associated with depressive symptoms

Multiple logistic regression analysis revealed that participants in the intervention group (adjusted odds ratio [AOR]: 0.68, 95% confidence interval [CI]: 0.52–0.88), those who had higher monthly household income per capita (AOR: 0.98, 95% CI: 0.97–0.99), and those who had higher self-esteem (AOR: 0.40, 95% CI: 0.26–0.62) had significantly lower odds of having depressive symptoms. On the other hand, women (AOR: 2.80, 95% CI: 1.92–4.07), those who had high internalized HIV-related stigma (AOR: 4.00, 95% CI: 2.77–5.76), and those who were food insecure (AOR: 3.44, 95% CI: 2.00–5.93) had significantly higher odds of having depressive symptoms (Table 2).

**Table 2:** Factors associated with depressive symptoms (*n*=674)

Variable	AOR (95% CI)	*p*
Program participation status		
Non-participants (ref.)	1.00	
Participants	**0.68 (0.52–0.88)**	**0.003**
Age (in years; mean=44)		
<44 (ref.)	1.00	
≥44	0.87 (0.59–1.28)	0.483
Gender		
Men (ref.)	1.00	
Women	**2.80 (1.92–4.07)**	**<0.001**
Formal education		
No (ref.)	1.00	0.635
Yes	0.93 (0.69–1.26)	
Marital status		
Divorced/separated/widowed/single (ref.)	1.00	
Married/cohabitating	0.82 (0.54–1.23)	0.332
Monthly household income per capita (in USD; median=20.6)		
<20.6 (ref.)	1.00	
≥20.6	**0.98 (0.97–0.99)**	**<0.001**
Debt in the past 12 months		
No (ref.)	1.00	
Yes	0.98 (0.68–1.40)	0.893
ART status		
Not receiving ART (ref.)	1.00	
Receiving ART	0.49 (0.04–5.75)	0.569
Time since HIV seropositive diagnosis (in years; median=8)		
<8 (ref.)	1.00	
≥8	0.71 (0.38–1.32)	0.281
Food assistance		
No (ref.)	1.00	
Yes	0.67 (0.37–1.21)	0.181
Internalized HIV-related stigma (median=3)		
Low (IA-RSS score 0–2) (ref.)	1.00	
High (IA-RSS score 3–6)	**4.00 (2.77–5.76)**	**<0.001**
Self-esteem (mean=28.7)		
Low (RSES score<28.7) (ref.)	1.00	
High (RSES score≥28.7)	**0.40 (0.26–0.62)**	**<0.001**
Household food insecurity		
No (ref.)	1.00	
Yes	**3.44 (2.00–5.93)**	**<0.001**

AOR, adjusted odds ratio; CI, confidence interval; ART, antiretroviral therapy; IA-RSS, Internalized AIDS-related Stigma Scale; RSES, Rosenberg Self-Esteem Scale. Bold denotes statistically significant AOR and *p*-value at 5% level.

### Impact of the livelihood program on depressive symptoms

A significant average treatment effect on the treated for depressive symptoms (T=−1.99) was detected when controlling for selection bias using propensity score matching (Table 3). Within this matched cohort, scores for depressive symptoms among the intervention group were significantly lower than those among the comparison group. After propensity score matching for depressive symptoms, all covariates except marital status were well balanced (i.e. less than 5% of standardized percentage bias [see Table 4] and a non-significant *p*-value). Rubin's B (7.0) and R (1.24) after matching were also sufficiently balanced (Table 5).

**Table 3:** Average treatment effect on the treated using propensity score matching

	Intervention group (*n*=356)	Comparison group (*n*=326)	Difference	SE	T-statistic
Unmatched	0.5618	0.6258	−0.0640	0.0376	−1.70
ATT	0.5618	0.6387	−0.0770	0.0387	−1.99

ATT, average treatment effect on the treated; SE, standard error.

**Table 4:** Comparison of balance between the intervention and comparison groups before and after propensity score matching for depressive symptoms (*n*=682)

		Mean				
					
Variable		Intervention group	Comparison group	% bias^a^	Reduction in % bias^a^	*p*
Age (in years; ≥44)	Unmatched	0.506	0.497	1.7		0.821
	Matched	0.506	0.502	0.7	60.2	0.927
Gender (women)	Unmatched	0.736	0.623	24.4		0.001
	Matched	0.736	0.717	4.2	82.9	0.562
Formal education (yes)	Unmatched	0.801	0.794	1.5		0.844
	Matched	0.801	0.800	0.1	93.1	0.989
Marital status (married/cohabitating)	Unmatched	0.567	0.629	−12.5		0.103
	Matched	0.567	0.596	−5.9	53.1	0.437
Monthly household income per capita	Unmatched	10.589	9.795	7.7		0.315
(in USD; ≥20.6)	Matched	10.589	10.685	−0.9	88.0	0.901
Debt in the past 12 months (yes)	Unmatched	0.744	0.718	6.0		0.434
	Matched	0.744	0.744	0.2	96.9	0.980
ART status (receiving ART)	Unmatched	0.978	0.979	−0.7		0.929
	Matched	0.978	0.980	−1.8	−163.3	0.808
Time since HIV seropositive diagnosis	Unmatched	0.573	0.537	7.3		0.342
(in years; ≥8)	Matched	0.573	0.569	0.8	89.2	0.916
Food assistance (yes)	Unmatched	0.379	0.463	−17.0		0.026
	Matched	0.379	0.374	0.9	94.8	0.904

ART, antiretroviral therapy.

a Standardized percentage bias: percentage of difference in sample means between the treated and non-treated sub-samples.

**Table 5:** Bias before and after propensity score matching

	Pseudo R^2^	LR *χ*^2a^	*p*	Mean bias	Median bias	Rubin's B^b^	Rubin's R^c^
Unmatched	0.022	20.82	0.013	8.8	7.3	35.2	0.98
Matched	0.001	0.88	1.000	1.7	0.9	7.0	1.24

a Likelihood-ratio chi-square test.

b Rubin's B: the absolute standardized difference of the means of the linear index of the propensity score between the treated and (matched) non-treated group.

c Rubin's R: the ratio of treated to (matched) non-treated variances in propensity score index.

## Discussion

This is the first study to assess the influence of a livelihood program on the mental health of people living with HIV in Cambodia. This study indicated a lower rate of depressive symptoms among livelihood program participants compared to comparison group participants. This is also one of the few studies reporting high rates of depressive symptoms among people living with HIV in Cambodia. The findings show that depressive symptoms were positively associated with several factors such as being female, having internalized HIV-related stigma, and suffering from food insecurity.

The analysis from propensity score matching indicated that the livelihood program might directly mitigate depressive symptoms among program participants. A qualitative study on a livelihood program conducted by KHANA in 2013 showed that village savings and loan associations allowed program participants to feel financially secure and independent because they had guaranteed access to money (37). In addition, program participants reported that their status in the community was elevated and stigma and discrimination toward them was reduced after they joined a village savings and loan association or received a cash grant (37). These findings are also consistent with findings of a qualitative study in Côte d'Ivoire, wherein village savings and loan associations played an important role in providing psychosocial support to association members, resulting in enhanced psychosocial well-being (57). This study also found that association members stopped expressing negative feelings such as hopelessness, loneliness, and anxiousness (57), all of which are related to depressive symptoms (58). Moreover, another qualitative study in Kenya demonstrated that a livelihood intervention positively changed not only participants’ self-esteem but also their status in the community (59). Considering the aforementioned results, participation in livelihood program activities may enhance economic self-confidence, community status, and self-worth and alleviate anxiety associated with economic hardship, food insecurity, stigma, and discrimination. Thus, the livelihood program may have an important role in mitigating the considerable burden of depressive symptoms experienced by people living with HIV in Cambodia.

Using the HSCL-25 cutoff, we identified a high prevalence (59.2%) of depressive symptoms among people living with HIV in Cambodia. This rate was 3.5 times as high as that of the general Cambodian population (16.7%) (23). In other countries, the rate of major depressive disorders among people living with HIV is two to four times that of HIV-negative populations (4, 5).

Compared to men, women had more than twice the odds (AOR: 2.80) of having depressive symptoms in this study. This result is consistent with previous findings indicating that the rate of depression for women was elevated in both the general population (23) and people living with HIV (3). As previous studies have shown, lower income per capita among HIV-affected households headed by widows and higher rates of harassment and physical violence among females living with HIV (34, 60) indicate that women might carry a heavier burden than men and might be more vulnerable to economic insecurity and mental disorders resulting from contracting HIV.

We also found that those who had higher internalized HIV-related stigma had four times higher odds of having depressive symptoms and that over half of the participants indicated higher internalized HIV-related stigma. Internalized HIV-related stigma has also been found to be associated with higher rates of depression in Thailand and South Africa (61, 62). Despite such stigmatization, there appear to be few interventions aimed at reducing internalized HIV-related stigma (63, 64). Thus, specific interventions are likely necessary for them, and livelihood programs may be one of the approaches to reducing internalized HIV-related stigma (59).

People living with HIV who had experienced food insecurity were more likely to have had depressive symptoms than were those who had experienced food security. In Burkina Faso, people who had experienced food insecurity reported feeling anxiety due to lack of sufficient food for their needs, shame from having to obtain food in socially unacceptable ways, and guilt because of an inability to feed their families (65, 66). Those feelings led to social deprivation and alienation, which in turn led to considerable mental and emotional distress (65, 67). Since both depressive symptoms and food insecurity are associated with poor adherence to ART as well as poorer clinical outcomes (52, 68), interventions for both conditions should be integrated into general HIV care programs. Food security is a basic human right, and it should not be threatened.

## Limitations

Although this study provides several novel findings, it has several limitations as well. First, this study adopted a quasi-experimental nonequivalent comparison group study design to assess the impact of the livelihood program on depressive symptoms among people living with HIV because baseline data were not available and a randomized design was not feasible. To overcome this weakness, propensity score matching were used to reduce potential selection biases between the intervention and comparison groups. Nevertheless, unobserved covariates could not be controlled because we lacked information on them (69), which means that selection bias could not be completely eliminated. A more rigorous research design is needed to examine the mechanisms and pathways for how livelihood programs affect depressive symptoms among people living with HIV.

Second, because we used self-report measures, over- and under-reporting might have occurred. Additionally, we measured depressive symptoms using the HSCL-25, which is only a screening tool; diagnostic confirmation of major depressive disorders was not conducted. This may have led to over- or under-estimation of depressive symptoms.

Third, we did not include some potentially important factors such as utilization of antidepressants or psychological therapies, which might influence the magnitude of depressive symptoms among people living with HIV. Future studies should consider such variables.

Finally, the Cronbach's alphas of the Internalized AIDS-Related Stigma Scale and Rosenberg Self-Esteem Scale (0.67 and 0.57, respectively) were low. This might have influenced the reliability of the study results.

## Conclusions

This study highlighted how KHANA's livelihood program may mitigate the burden of depressive symptoms among people living with HIV in Cambodia. Nevertheless, this program could be modified to better emphasize participants’ mental health and should be expanded to areas where it has not yet been implemented in the name of social fairness and ethics. Donor agencies might consider providing further financial support to help extend the coverage of the program through KHANA and its implementing partners. In addition, interventions specifically targeting women and those with lower household incomes should be implemented along with greater efforts to reduce stigma against people living with HIV.

These findings are valuable for both governmental institutions and NGOs for implementing more effective livelihood programs for people living with HIV. Furthermore, the main results of this study that livelihood programs may mitigate the burden of depressive symptoms could be used to inform, improve, and expand current activities. This is particularly crucial given that the majority of people living with HIV in this study experienced depressive symptoms.

## Paper context

Past research has examined the potential impact of livelihood programs on food insecurity, HIV morbidity, and stigma among HIV-positive people. In this study, people living with HIV who attended a livelihood program were less likely to have depressive symptoms compared with those who did not attend it. Similar livelihood programs should be implemented on a larger scale to help mitigate the mental health problems of people living with HIV.
